# Structural and functional imaging features of cognitive phenotypes in pediatric multiple sclerosis

**DOI:** 10.1002/acn3.52090

**Published:** 2024-05-28

**Authors:** Damiano Mistri, Monica Margoni, Elisabetta Pagani, Paola Valsasina, Alessandro Meani, Lucia Moiola, Massimo Filippi, Maria A. Rocca

**Affiliations:** ^1^ Neuroimaging Research Unit, Division of Neuroscience IRCCS San Raffaele Scientific Institute Milan Italy; ^2^ Neurorehabilitation Unit IRCCS San Raffaele Scientific Institute Milan Italy; ^3^ Neurology Unit IRCCS San Raffaele Scientific Institute Milan Italy; ^4^ Neurophysiology Service IRCCS San Raffaele Scientific Institute Milan Italy; ^5^ Vita‐Salute San Raffaele University Milan Italy

## Abstract

**Objective:**

The present study aimed to identify the clinical and MRI features of the distinct cognitive phenotypes in pediatric multiple sclerosis (pedMS).

**Methods:**

PedMS patients (*n* = 73) and healthy controls (*n* = 30) underwent clinical examination and 3.0T MRI. All patients completed neuropsychological testing, and cognitive phenotypes were identified by performing *K*‐means clustering on cognitive scores. MRI metrics included brain T2‐hyperintese lesion volume and normalized brain volumes. Within seven cognitively relevant cortical networks, structural disconnectivity (i.e., the mean percentage of streamlines connecting each pair of cortical regions passing through a lesion) and resting‐state (RS) functional connectivity (FC) were estimated.

**Results:**

Three cognitive phenotypes emerged: Preserved cognition (PC; *n* = 27, 37%), mild verbal learning and memory/semantic fluency involvement (MVS; *n* = 28, 38%), and multidomain involvement (MI; *n* = 18, 25%). Age, sex, and disease duration did not differ among groups. Compared with healthy subjects, PC patients had decreased RS FC within the default mode network (*p* = 0.045); MVS patients exhibited lower cortical volume and reduced RS FC within the frontoparietal network (all *p* = 0.045); and MI patients showed decreased volumes in all brain compartments except the hippocampus, and reduced RS FC within the frontoparietal network (all *p* ≤ 0.045). Compared to PC, MI patients had more severe disability and higher structural disconnectivity within four cortical networks (all *p* ≤ 0.045). Compared to PC and MVS, MI patients had lower intelligence quotient (all *p* ≤ 0.005).

**Interpretation:**

We identified three cognitive phenotypes in pedMS that demonstrate the existence of a spectrum of impairment. Such phenotypes showed distinct clinical and MRI characteristics that contributed to explain their cognitive profiles.

## Introduction

Pediatric multiple sclerosis (MS) accounts for approximately 2%–10% of all MS cases.^1^ Despite having higher relapse rate than the adult counterparts, pediatric patients exhibit a slower transition to secondary progressive MS and a more gradual development of physical disability.^1^ However they tend to experience a steeper cognitive decline, leading to cognitive dysfunction at a significantly younger age in more than 30% of cases.^1^, ^2^ Processing speed, complex aspects of attention and memory are the most commonly impaired cognitive functions. Unlike adults, language abilities and general intelligence may also be affected.^3^

MRI studies in pediatric MS have shown that a higher frequency of focal white matter (WM) lesions in the corpus callosum, thalamus, and parieto‐occipital regions,^4^ as well as more severe atrophy of the these regions^5^ and the hippocampus^6^ contribute to cognitive dysfunction. Moreover, patients with cognitive impairment exhibit reduced resting‐state (RS) functional connectivity (FC) of the precuneus^4^, ^7^ and dentate nucleus with basal ganglia, frontal, temporal, and parietal regions, compared to cognitively preserved patients.^8^

Most previous investigations classified cognitive functioning according to a dichotomous view, namely, preserved versus impaired, overlooking the heterogeneity of cognitive manifestations in pediatric MS.^9^ Recent studies in adult MS patients^9^, ^10^ deployed machine learning models to identify the recurring patterns of cognitive deficit and demonstrated the existence of four to five cognitive phenotypes ranging from intact cognition to severe and widespread impairments in cognitive funcioning.^9^, ^10^ Work extending this cognitive phenotypes framework beyond adult‐onset MS is lacking. A detailed description of the different cognitive profiles in children and adolescents with MS could offer a deeper understanding of the neural substrates of pediatric MS‐related cognitive abnormalities, potentially providing the basis for more tailored interventions.

Against this background, in this study we employed an unsupervised machine learning technique to determine whether unique cognitive phenotypes exist in pediatric MS. Then, we investigated the association between these cognitive phenotypes and specific demographic, clinical, and MRI variables, encompassing brain T2‐hyperintese lesion volume (LV), normalized brain volumes, as well as structural disconnectivity (i.e., the mean proportion of connecting streamlines passing through a lesion estimated using the Network Modification [NeMo] Tool)^11^, ^12^ and RS FC strength within seven main cortical networks (visual network, somatomotor network, dorsal attention network, ventral attention network, limbic network, frontoparietal network, and default mode network).^13^

## Methods

### Participants

This retrospective cross‐sectional study included data from 73 relapsing–remitting^14^ pediatric MS patients and 30 age‐, sex‐and education‐matched healthy controls (HCs) recruited at the IRCCS San Raffaele Scientific Institute in Milan, Italy. Inclusion criteria: no previous history of major systemic, psychiatric, or neurological disorders (other than MS); no concomitant therapy with antidepressants or psychoactive drugs; right‐handedness.^15^ In addition, pediatric MS patients were required to be relapse‐ and steroid‐free for at least 1 month before the clinical and MRI examination; and to have a score < 20 on the Children's Depression Inventory, which represents the clinical threshold for depressive symptoms.^16^ Appropriate testing was performed as necessary to rule out leukodystrophies and myelin oligodendrocyte glycoprotein antibody‐associated disorders.

### Clinical and neuropsychological assessment

On the day of the MRI, all patients underwent neurological examination with Expanded Disability Status Scale (EDSS)^17^ assessment and record of ongoing disease‐modifying treatments. Experienced neuropsychologists administered the Brief Neuropsychological Battery for Children^18^ which assesses verbal learning and memory (using the Selective Reminding Test), visuospatial learning and memory (through the 10/36 Spatial Recall Test), attention and information processing speed (using the Trail Making Test and the Symbol Digit Modalities Test), and expressive language (through a Semantic verbal fluency test and a Phonemic verbal fluency test). Z‐scores were calculated for each cognitive test according to normative data.^18^ A z‐score for each cognitive domain was then obtained by averaging the z‐scores of the corresponding tests.^19^ For patients in the age range between 6 and 15 years, the intelligence quotient was assessed using the Wechsler Intelligence Scale for Children,^20^ while for patients with age ≥ 16 years it was measured with the Wechsler Adult Intelligence Scale.^21^ Fatigue was also evaluated through the Fatigue Severity Scale (FSS).^22^

### MRI acquisition

Brain images were acquired from all subjects using two 3.0 T MRI scanners (first scanner: Achieva [*n* = 21 HCs and *n* = 55 pediatric MS patients], second scanner: Ingenia [*n* = 9 HCs and *n* = 17 pediatric MS patients] Philips Medical Systems, Eindhoven, The Netherlands). Images acquired using the Achieva scanner included (1) T2*‐weighted single shot echo planar imaging (EPI) sequence for RS functional MRI (fMRI) (repetition time [TR] = 3000 ms, echo time [TE] = 35 ms, field of view [FOV] = 240 mm^2^, matrix size = 128 × 128, flip angle [FA] = 90°, 30 contiguous axial slices, 4 mm thick); (2) dual‐echo turbo spin echo (TR = 2599, TE = 16–80 ms, FOV = 240 mm^2^, FA = 90°, echo train length [ETL] = 6, matrix = 256 × 256, 44 contiguous axial slices, 3 mm thick); and (3) 3D T1‐weighted fast field echo (TR = 25, TE = 4.6 ms, FOV = 230 mm^2^, FA = 30°, matrix size = 256 × 256, 220 contiguous axial slices, 0.8 mm thick). Images acquired using the Ingenia scanner included: (1) T2*‐weighted EPI sequence for RS fMRI, with the same parameters used for the Achieva scanner (TR = 3000 ms, TE = 35 ms, matrix size = 128 × 128, FA = 90°, FOV = 240 mm^2^, 30 contiguous axial slices, 4 mm thick); (2) 3D fluid‐attenuated inversion recovery (FLAIR; TR = 4800 ms, inversion time [TI] = 1650 ms, TE = 270 ms, matrix size = 256 × 256, FOV = 256 × 256 mm^2^, ETL = 167, 192 contiguous sagittal slices, 1 mm thick); (3) 3D T2‐weighted sequence (TR = 2500 ms, TE = 330 ms, ETL = 117, FOV = 256 × 256 mm^2^, matrix size = 256 × 256, 192 contiguous sagittal slices, 1 mm thick); and (4) 3D T1‐weighted turbo field echo (TR = 7 ms, TI = 1000 ms, FA = 8°, TE = 3.2 ms, FOV = 256 × 256 mm^2^, matrix size = 256 × 256, 204 contiguous sagittal slices, 1 mm thick).

### Conventional MRI analysis

An experienced observer manually outlined T2‐hyperintense WM lesions from the dual‐echo scans of pediatric MS patients acquired with the Achieva scanner. Lesion volume (LV) was then calculated using a semi‐automated local thresholding segmentation technique (Jim 7.0, Xinapse Systems Ltd, Colchester, UK). For patients acquired on the Ingenia scanner, FLAIR images were resampled at the same resolution of dual‐echo images, lesions were manually segmented as described above, and total T2‐LV was measured. On both scanners, normalized brain, WM, and cortical GM volumes were calculated using FSL SIENAx software on lesion‐filled 3D T1‐weighted images.^23^ Normalized volumes of bilateral thalamus and bilateral hippocampus were also measured with the FIRST tool.^24^

### Structural disconnection analysis

Lesion masks were transformed onto the Montreal Neurologic Institute (MNI) space using the nonlinear transformation that had been previously calculated for the SIENAx software. The spatially transformed lesion maps were uploaded to the NeMo tool (https://kuceyeski‐wcm‐web.s3.us‐east‐1.amazonaws.com/upload.html),^11^ a stand‐alone web application based on a set of diffusion‐weighted image scans of 420 healthy subjects from the 7T Human Connectome Project. We selected the following options: constrained spherical‐deconvolution informed filtering of tractograms^25^; the CocoYeo243‐subj parcellation with 243 regions obtained from Schaefer200 (200 cortical), aseg (16 subcortical), and SUIT (27 cerebellar).^26^ The NeMo tool computed the average pairwise disconnection (i.e., change in connectivity score, which is the proportion of WM streamlines that intersect the volume occupied by a lesion; 0 = no disconnection, 1 = complete disconnection)^11^ across the reference sample and displayed the results into a symmetrical *N* × *N* matrix, where *N* represents the number of atlas regions. Finally, we calculated disconnection between regions of seven cortical brain networks (visual network, somatomotor network, dorsal attention network, ventral attention network, limbic network, frontoparietal network, and default mode network, according to the Yeo atlas^13^) and derived an average disconnection value for each network.

### RS FC network preprocessing

The CONN toolbox^27^ was utilized to process RS fMRI data. Using a rigid body transformation, images were first realigned to the mean of each session to correct for head movements. The mean framewise displacement was calculated as a measure of motion. Subsequently, RS fMRI images were registered to the lesion filled 3D T1‐weighted scan, normalized to the Montreal Neurological Institute space using a nonlinear transformation, and smoothed with a 6‐mm^3^ Gaussian filter. Denoising was performed using the first five cerebrospinal fluid and WM principal components as nuisance covariates through the anatomical component‐based noise correction method.^28^ The six rigid movement parameters and their first temporal derivatives were regressed out from the data, along with any outliers and spurious effects from the first two time points identified by the ART toolbox. Finally, the RS fMRI time series were linearly detrended and band‐pass filtered between 0.01 and 0.1 Hz.

### RS FC network analysis

After RS fMRI preprocessing, the brain was parceled into 200 cortical regions of interest (ROI) according to the parcellation proposed by Schaefer et al.^26^ RS fMRI time series were extracted from each region by calculating the mean signal of all voxels within each ROI. Bivariate correlations between each pair of ROI, representing the RS FC strengths between cortical regions, were calculated with the Pearson's correlation coefficient between ROI time courses. Correlation matrices were obtained from all participants and were thresholded at correlation threshold *τ* = 0 into weighted connectivity matrices. As measure of RS FC strength, we used the degree, that is, the weighted sum of connections for each node (i.e., cortical ROI). Finally, the average degree within seven cortical networks (according to the parcellation proposed by Yeo et al.^13^) was calculated.

### Statistical analysis

Demographic and clinical characteristics were compared between HC and pediatric MS patients using the Mann–Whitney *U* test and Chi‐square test, as appropriate. For each participant, normalized brain volumes z‐scores and RS FC degrees z‐scores were calculated by subtracting the mean and dividing by the standard deviation of the HCs scanned on the same scanner. Brain T2 LVs were log‐transformed and then standardized into z‐scores according to the overall distribution observed in pediatric MS patients.

A *K*‐means cluster analysis was conducted on z‐scores of neuropsychological tests to detect homogeneous cognitive phenotypes in pediatric MS patients. The *K*‐means algorithm partitions the data into distinct groups to minimize within‐cluster variance.^29^ To increase the likelihood of obtaining a reliable clustering solution, the procedure was iterated 50 times, thus ensuring greater robustness and reducing bias associated with the random selection of centroids at the start of the algorithm. The NBclust R package^30^ was used to determine the optimal number of clusters through a consensus voting approach across 23 different indices.

Demographic, clinical, and neuropsychological differences between cognitive phenotypes were examined using the Chi‐square test, Mann–Whitney *U* test, or linear models. Benjamini–Hochberg false discovery rate (FDR) correction was applied to account for multiple testing. Comparisons of MRI variables between HC and cognitive phenotypes were performed using age‐ and sex‐adjusted linear models, applying FDR correction.

Statistical significance was set at *p* < 0.05. R‐4.2.2 and SPSS version 26.0 (IBM, Armonk, NY, USA) software were used for computations.

## Results

### Demographic, clinical, and conventional MRI measures

Participants' demographics are summarized in Table 1. Compared with HCs, pediatric MS patients had reduced normalized brain volume (*p* = 0.002), normalized cortical GM volume (*p* = 0.002), and normalized thalamic volume (*p* = 0.024) (Table 1).

**Table 1:** Main demographic, clinical, and conventional MRI characteristics of the subjects enrolled in the study.

	HC (*n* = 30)	pedMS patients (*n* = 73)	*p*
Girls, No (%)	15 (50)	49 (67)	0.16^a^
Age, median (IQR), y	15.2 (12.0 to 18.0)	15.9 (14.2 to 17.0)	0.63^b^
Education, median (IQR), y	8.0 (7.0 to 11.0)	9.0 (8.0 to 11.0)	0.64^b^
EDSS, median (IQR)	NA	1.0 (1.0 to 1.5)	NA
Disease duration, median (IQR), y	NA	1.4 (0.7 to 2.6)	NA
CDI, median (IQR)	NA	6.0 (3.0 to 10.0)	NA
FSS, mean (SD)	NA	2.7 (1.1)	NA
Patients receiving DMTs: No treatment, first line, second line, No. (%)^†^		11 (15)/37 (51)/25 (34)	NA
Subjects scanned with: S1, S2, No (%)	21 (70)/9 (30)	55 (75)/17 (25)	0.67^a^
z‐T2 lesion volume, mean (SD)	NA	0.0 (1.0)	NA
z‐N brain volume, mean (SD)	**0.0 (1.0)**	**−0.8 (1.1)**	**0.002** ^c^
z‐N WM volume, mean (SD)	0.0 (1.0)	−0.3 (1.4)	0.37^c^
z‐N cortical GM volume, mean (SD)	**0.0 (1.0)**	**−0.6 (0.9)**	**0.002** ^c^
z‐N hippocampal volume, mean (SD)	0.0 (1.0)	−0.1 (1.0)	0.26^c^
z‐N thalamic volume, mean (SD)	**0.0 (1.0)**	**−0.7 (1.3)**	**0.024** ^c^

Bold text indicates a statistically significant result.

CDI, Children's Depression Inventory; DMT, disease‐modifying treatment; EDSS, Expanded Disability Status Scale; GM, gray matter; FSS, Fatigue Severity Scale; HC, healthy controls; IQR, interquartile range; N, normalized; pedMS, pediatric multiple sclerosis; S, scanner; SD, standard deviation; WM, white matter; z, z‐score.

^a^ Chi‐square test.

^b^ Mann–Whitney *U* test.

^c^ Age‐ and sex‐adjusted linear model.

^†^ Classification of DMTs: first line = interferon beta and glatiramer acetate; second line = natalizumab, rituximab, fingolimod, cyclophosphamide, and mitoxantrone.

### Cognitive phenotypes

**Figure 1:**
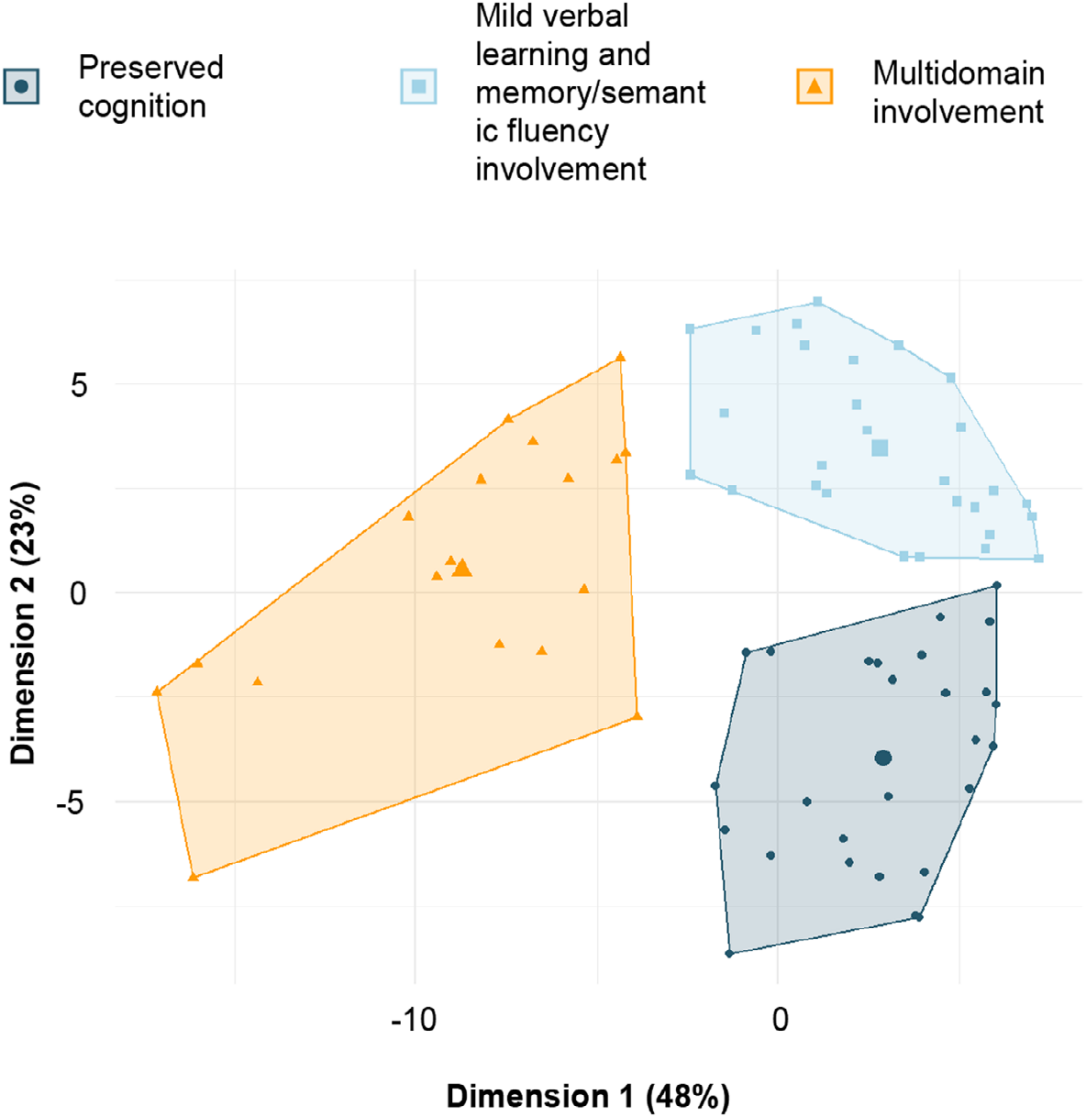
Cluster plots of the three cognitive phenotypes identified from cognitive tests z‐scores. Circles represent pediatric MS patients with preserved cognition, squares—patients with mild verbal learning and memory/semantic fluency involvement, and triangles—patients with multidomain involvement. The big circle, square, and triangle represent the centroids for each cluster.

**Figure 2:**
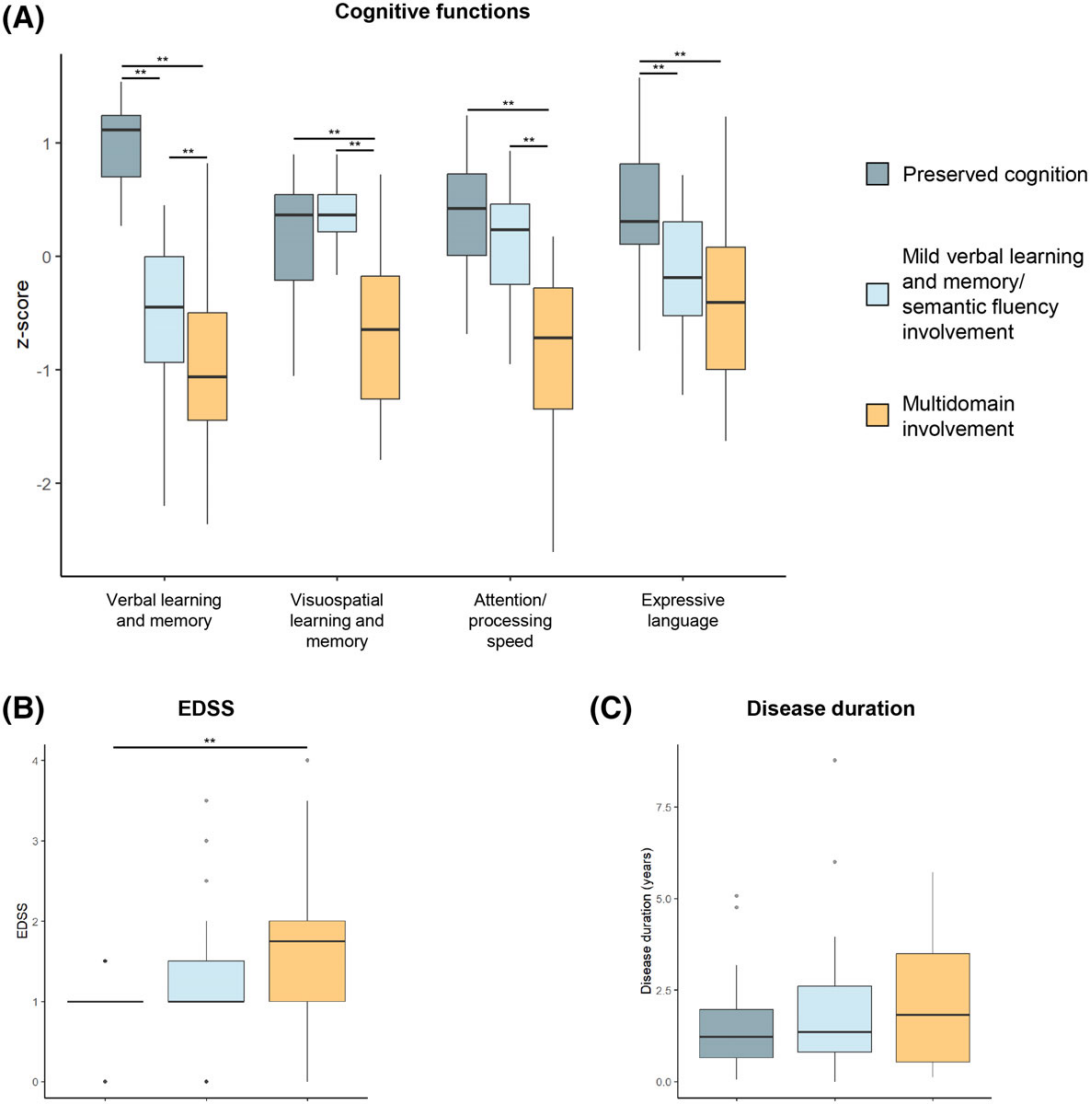
Comparisons of cognitive and clinical variables between cognitive phenotypes. Boxplot of cognitive domain z‐scores (A), Expanded Disability Status Scale (EDSS) (B) and disease duration (C) in pediatric MS patients grouped by cognitive phenotype. For each variable the horizontal line represents the median. Asterisks indicate statistically significant differences (**p < 0.01; *p < 0.05).

The optimal number of clusters was three as suggested by the majority rule (13 out of 23 indices).^30^ Pediatric MS patients were then subdivided into three cognitive phenotypes by *K*‐means clustering analysis (Fig. 1). The first cluster “preserved cognition” (PC) included 27 patients (37%) who showed a pattern of average or above‐average functioning across cognitive domains and a median intelligence quotient of 104 (interquartile range [IQR], 100–118). The second group “mild verbal learning and memory/semantic fluency involvement” (MVS) comprised 28 patients (38%). The median intelligence quotient was 100 (IQR, 92–117). The third group was named “multidomain involvement” (MI) and included 18 patients (25%) who had below‐average to exceptionally low scores on all cognitive tests. The intelligence quotient was lower in MI (median [IQR] intelligence quotient, 92 [76–96]; FDR‐p ≤ 0.005) than in MVS and PC patients (Table 2). No significant differences were found when comparing age, sex, education, disease duration, and FSS scores among cognitive phenotypes (FDR‐p ≥ 0.44) (Fig. 2). Patients with MI (median [IQR] EDSS score, 1.5 [1.0–2.0]) had more severe physical disability than those with PC (median [IQR] EDSS score, 1.0 [0.0–1.5]; FDR‐p = 0.006) (Table 2) (Fig. 2).

**Table 2:** Main demographic, clinical, and neuropsychological features of cognitive phenotypes.

	PC (*n* = 27)	MVS (*n* = 28)	MI (*n* = 18)	FDR‐p
Girls, No (%)	21 (78)	17 (61)	11 (61)	≥0.44
Age, median (IQR), y	15.9 (13.7 to 16.8)	16.1 (14.5 to 17.0)	15.7 (14.1 to 17.0)	≥0.99
Education, median (IQR), y	9.0 (8.0 to 11.0)	10.0 (8.0 to 11.0)	9.0 (8.0 to 11.0)	≥0.44
EDSS, median (IQR)	1.0 (0.0 to 1.5)	1.0 (1.0 to 1.5)	1.5 (1.0 to 2.0)	**0.006** ^a^
Disease duration, median (IQR), y	1.2 (0.6 to 2.1)	1.4 (0.8 to 2.6)	1.8 (0.5 to 3.6)	≥0.99
CDI, median (IQR)	7.5 (3.0 to 12.0)	5.5 (3.0 to 8.0)	6.0 (1.5 to 11.0)	≥0.90
FSS, mean (SD)	2.7 (0.8)	2.7 (1.2)	2.9 (1.3)	≥0.99
Patients receiving DMTs: No treatment, first line, second line, No. (%)^†^	3 (11)/16 (59)/8 (30)	5 (18)/16 (57)/7 (25)	3 (17)/5 (28)/10 (55)	NA
IQ, median (IQR)	104 (100 to 118)	100 (92 to 117)	92 (76 to 96)	**≤0.005** ^a,b^
z‐SRT‐lts, mean (SD)	1.0 (0.5)	−0.4 (0.8)	−1.2 (1.1)	**≤0.005** ^a,b,c^
z‐SRT‐cltr, mean (SD)	0.7 (0.8)	−0.7 (0.7)	−1.2 (1.0)	**<0.001** ^a,c^
z‐SRT‐recall, mean (SD)	1.0 (0.5)	−0.5 (1.0)	−1.2 (1.3)	**<0.001** ^a,c^
z‐SPART, mean (SD)	0.4 (1.2)	0.6 (0.8)	−1.8 (2.0)	**<0.001** ^a,b^
z‐SPART‐recall, mean (SD)	0.4 (0.8)	0.5 (0.7)	−2.0 (1.6)	**<0.001** ^a,b^
z‐TMT‐A, mean (SD)	0.2 (0.7)	0.1 (0.7)	−0.9 (1.0)	**<0.001** ^a,b^
z‐TMT‐B, mean (SD)	0.2 (0.7)	0.2 (0.7)	−1.2 (1.5)	**<0.001** ^a,b^
z‐SDMT, mean (SD)	0.4 (1.0)	0.0 (0.6)	−0.5 (0.6)	**<0.001** ^a^
z‐Semantic fluency test, mean (SD)	0.4 (0.8)	−0.3 (0.4)	−0.6 (0.5)	**<0.001** ^a,c^
z‐Phonemic fluency test, mean (SD)	0.3 (1.0)	0.0 (0.7)	−0.2 (1.2)	≥0.12
z‐Verbal learning and memory, mean (SD)	0.9 (0.5)	−0.6 (0.7)	−1.2 (1.1)	**≤0.012** ^a,b,c^
z‐Visuospatial learning and memory, mean (SD)	0.2 (0.6)	0.3 (0.4)	−0.6 (0.8)	**<0.001** ^a,b^
z‐Attention/processing speed, mean (SD)	0.3 (0.6)	0.1 (0.6)	−0.9 (0.8)	**<0.001** ^a,b^
z‐Expressive language, mean (SD)	0.4 (0.7)	−0.2 (0.5)	−0.4 (0.7)	**≤0.005** ^a,c^

Comparisons performed by Chi‐square test (sex), linear models (age, IQ and cognitive z‐scores), and Mann–Whitney *U* test (education, EDSS, disease duration, and CDI). FDR correction was applied to account for the overall number of tests. Letters indicate significant differences as follows: ^a^PC versus MI; ^b^MVS versus MI; ^c^PC versus MVS. Bold text indicates a statistically significant result.

CDI, Children's Depression Inventory; cltr, consistent long‐term retrieval; EDSS, Expanded Disability Status Scale; FDR, false discovery rate; IQ, intelligence quotient; lts, long term storage; MI, multidomain involvement; MVS, mild verbal learning and memory/semantic fluency involvement; PC, preserved cognition; SD, standard deviation; SDMT, Symbol Digit Modalities Test; SPART, Spatial Recall Test; SRT, Selective Reminding Test; TMT, Trail Making Test; z, z‐score.

^†^ Classification of DMTs: first line = interferon beta and glatiramer acetate; second line = natalizumab, rituximab, fingolimod, cyclophosphamide, and mitoxantrone.

### MRI features of cognitive phenotypes

#### Conventional MRI features

The comparisons of normalized brain volumes between HC and pediatric MS patients with PC revealed no statistically significant differences. Compared with HC, patients with MVS had lower normalized cortical GM volume (FDR‐p = 0.045), while those with MI showed lower normalized brain (FDR‐p < 0.001), WM (FDR‐p = 0.045), cortical GM (FDR‐p = 0.010), and thalamic volumes (FDR‐p = 0.041). Compared with patients with PC, those with MI had lower normalized brain volume (FDR‐p = 0.037). When compared to patients with MVS those with MI were characterized by lower normalized brain volume (FDR‐p = 0.044) and normalized WM volume (FDR‐p = 0.045). No significant differences were found between PC and MVS phenotypes (Table 3).

**Table 3:** Differences in estimated marginal means of MRI variables across cognitive phenotypes.

	HC (*n* = 30)	PC (*n* = 27)	MVS (*n* = 28)	MI (*n* = 18)	FDR‐p
z‐T2 lesion volume	NA	0.3 (−0.1 to 0.7)	0.1 (−0.3 to 0.5)	−0.4 (−0.9 to 0.1)	≥0.12
z‐N brain volume	−0.1 (−0.4 to 0.3)	−0.4 (−0.8 to −0.1)	−0.6 (−1.0 to −0.3)	−1.4 (−1.8 to −0.9)	**≤0.044** ^a,b,c^
z‐N WM volume	0.0 (−0.5 to 0.5)	0.0 (−0.5 to 0.4)	0.0 (−0.4 to 0.5)	−1.1 (−1.7 to −0.5)	**0.045** ^a,c^
z‐N cortical GM volume	0.0 (−0.4 to 0.2)	−0.4 (−0.7 to −0.1)	−0.6 (−0.9 to −0.3)	−1.0 (−1.4 to −0.6)	**≤0.045** ^a,d^
z‐N hippocampal volume	0.0 (−0.4 to 0.4)	−0.1 (−0.5 to 0.3)	−0.2 (−0.6 to 0.2)	−0.5 (−1.0 to 0.0)	≥0.25
z‐N thalamic volume	0.0 (−0.5 to 0.4)	−0.4 (−0.9 to 0.1)	−0.5 (−1.0 to −0.1)	−1.2 (−1.8 to −0.7)	**0.041** ^a^
Structural disconnectivity					
Visual network	NA	0.2 (0.1 to 0.3)	0.2 (0.1 to 0.3)	0.3 (0.2 to 0.4)	≥0.10
Somatomotor network	NA	0.2 (0.1 to 0.3)	0.2 (0.2 to 0.3)	0.3 (0.3 to 0.4)	**0.041** ^b^
Dorsal attention network	NA	0.2 (0.1 to 0.3)	0.2 (0.2 to 0.3)	0.4 (0.3 to 0.5)	**0.045** ^b,c^
Ventral attention network	NA	0.2 (0.1 to 0.3)	0.2 (0.2 to 0.3)	0.3 (0.3 to 0.4)	≥0.07
Limbic network	NA	0.1 (0.0 to 0.1)	0.1 (0.0 to 0.1)	0.1 (0.1 to 0.2)	**0.046** ^b^
Frontoparietal network	NA	0.2 (0.1 to 0.3)	0.2 (0.2 to 0.3)	0.3 (0.3 to 0.4)	**0.045** ^b^
Default mode network	NA	0.2 (0.1 to 0.3)	0.2 (0.2 to 0.3)	0.3 (0.3 to 0.4)	≥0.07
RS FC z‐degree					
Visual network	0.1 (−0.3 to 0.6)	0.3 (0.0 to 0.7)	0.1 (−0.3 to 0.5)	0.3 (−0.1 to 0.8)	≥0.80
Somatomotor network	0.0 (−0.4 to 0.4)	0.2 (−0.2 to 0.5)	0.1 (−0.3 to 0.5)	0.1 (−0.3 to 0.5)	≥0.89
Dorsal attention network	0.0 (−0.3 to 0.4)	−0.1 (−0.4 to 0.2)	0.0 (−0.3 to 0.3)	−0.1 (−0.5 to 0.3)	≥0.88
Ventral attention network	0.1 (−0.4 to 0.5)	−0.2 (−0.7 to 0.2)	−0.4 (−0.9 to 0.0)	−0.3 (−0.8 to 0.2)	≥0.32
Limbic network	0.0 (−0.3 to 0.5)	0.0 (−0.3 to 0.4)	0.0 (−0.3 to 0.4)	0.0 (−0.5 to 0.4)	≥0.90
Frontoparietal network	0.1 (−0.3 to 0.4)	−0.5 (−0.8 to −0.2)	−0.6 (−0.9 to −0.3)	−0.7 (−1.1 to −0.4)	**0.045** ^a,d^
Default mode network	0.1 (−0.3 to 0.4)	−0.6 (−0.9 to −0.3)	−0.4 (−0.7 to −0.1)	−0.3 (−0.7 to 0.0)	**0.045** ^e^

Comparisons performed by age‐ and sex‐adjusted linear models. FDR correction was applied to account for the overall number of tests. Letters indicate significant differences as follows: ^a^HC versus MI; ^b^PC versus MI; ^c^MVS versus MI; ^d^HC versus MVS; ^e^HC versus PC. Data are presented as estimated marginal means (95% confidence interval). Bold text indicates a statistically significant result.

FDR, false discovery rate; GM, gray matter; HC, healthy controls; LV, lesion volume; MI, multidomain involvement; MVS, mild verbal learning and memory/semantic fluency involvement; N, normalized; PC, preserved cognition; RS FC, resting‐state functional connectivity; WM, white matter; z, z‐score.

#### Structural disconnectivity measures

**Figure 3:**
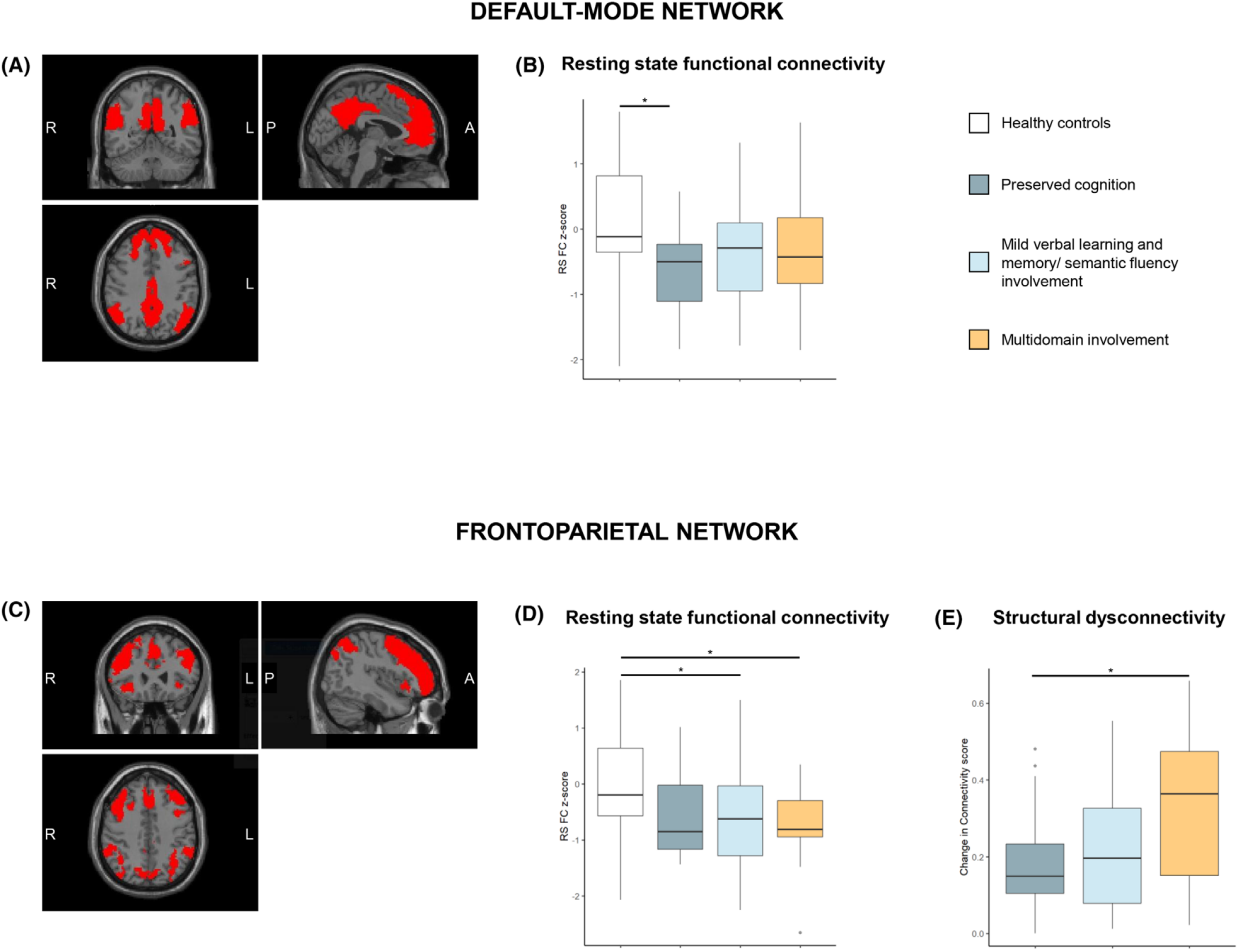
Differences in structural and functional MRI metrics between cognitive phenotypes. (A) Illustrates the default mode network. (B) Boxplot of participants' resting‐state (RS) functional connectivity (FC) degree within the default mode network. (C) Illustrates the frontoparietal network. Boxplot of participants' RS FC degree (D) and structural disconnectivity (E) within the frontoparietal network. For each variable the horizontal line represents the median. Asterisks indicate statistically significant differences (**p < 0.01; *p < 0.05).

No significant differences in metrics of structural disconnectivity were found between pediatric MS patients with PC and those with MVS. Structural disconnection within the dorsal attention network was significantly more severe in MI patients compared with patients with PC (FDR‐p = 0.045) and MVS (FDR‐p = 0.045). Additionally, when compared to the PC phenotype, the MI phenotype demonstrated more severe structural disconnectivity within the somatomotor (FDR‐p = 0.045), limbic (FDR‐p = 0.046), and frontoparietal (FDR‐p = 0.045) networks (Table 3) (Fig. 3).

#### RS FC network differences across cognitive phenotypes

Compared with HC, pediatric MS patients with PC exhibited significantly lower RS FC within the default mode network (FDR‐p = 0.045) (Fig. 3), while patients with MVS (FDR‐p = 0.045) and MI (FDR‐p = 0.045) showed lower mean RS FC z‐degree within the frontoparietal network (Fig. 3). No significant differences in RS FC z‐degree were observed among cognitive phenotype in any of the seven networks analyzed (Table 3).

## Discussion

By employing the *K*‐means clustering algorithm, we identified three distinct cognitive phenotypes corresponding to different degrees of impairment in patients with pediatric MS. Using such an approach, in addition to patients showing intact cognition or global cognitive decline, we could identify a third cognitive profile characterized by mild deficits in verbal learning and memory and semantic fluency. By analyzing structural and functional MRI network metrics, we were able to define the neural substrates of each cognitive phenotype, providing biological evidence to support our classification.

In this study, we observed a prevalence of cognitive impairment in 63% of pediatric MS patients, exceeding the rates of 22%–35% reported in previous studies.^3^ This result may be related to the presence of patients exhibiting subtle deficits in verbal learning and memory and semantic fluency (*n* = 28; 38%) that may elude the current classification of cognitive impairment.

We identified three cognitive phenotypes in a group of 73 pediatric MS patients through the application of *K*‐Means cluster analysis. The first cognitive phenotype, PC (37% of the sample), showed the most intact cognitive profile and was characterized by a lower level of physical disability and a higher intelligence quotient than patients with the most impaired neuropsychological profile, namely MI. MRI studies performed on healthy children and adolescents demonstrated a positive correlation between general intelligence and WM integrity,^31^ as well as higher cortical thickness in the frontal, temporal, parietal and occipital regions.^31^ Previous research in pediatric MS showed that higher intelligence quotient was associated with preservation of GM volume in the precuneus, cingulate cortex, frontal and temporal regions.^32^ In line with these results, our investigation revealed no significant differences in normalized brain volumes between HCs and the PC phenotype, suggesting that brain structural abnormalities in these patients are limited. On the other hand, PC patients had a significant reduction of RS FC within the default mode network, a result consistent with a previous study showing decreased RS FC between the default mode network and the visual network in pediatric MS patients with no or minimal disability.^33^ Studies on adult MS patients^34^ have shown that WM damage may determine reduced RS FC within the default mode network in the first phases of the disease, whereas, as disease progresses, RS FC may increase and reflect a maladaptive process potentially contributing to cognitive decline.^35^ In our study, no significant differences in default mode network RS FC were observed between HC and pediatric MS patients with MVS and MI. In such cases, it is tempting to speculate that the two effects (decreased default mode network connectivity due to MS‐related WM damage and increased RS FC, indicating maladaptive processes) outweighed each other, resulting in no detectable changes. However, caution should be used in interpreting these findings as previous investigations reported both increased^36^ and decreased^4^ FC in default mode network associated with cognitive impairment in pediatric MS.

The second cluster included 28 patients (38% of the sample) with mild deficits in verbal learning and semantic fluency. Of note, two large cross‐sectional investigations^9^, ^10^ identified a similar cognitive phenotype in adult MS patients. One possible explanation for the co‐occurrence of these deficits is that the ability to retrieve verbal information from long‐term memory, which is required for both verbal fluency and verbal memory tasks,^37^ was mildly impaired in patients belonging to this cognitive phenotype. This hypothesis is supported by the findings of a previous study^38^ in which episodic memory dysfunction emerged as one of the most important predictors of semantic fluency impairment in MS. However, semantic fluency is a multifactorial task that also relies on executive function, attention and language abilities,^39^, ^40^ and this process can fail due to deficits in any of these cognitive components. Furthermore, while early works on memory impairment in MS posited that difficulty retrieving information from long‐term memory was the primary cause of this deficit,^41^ subsequent studies suggested that the primary problem lies in the initial learning of information.^41^

Verbal fluency develops during childhood and adolescence along with ongoing brain maturation,^42^ but research into its neural correlates remains relatively limited in children.^42^ Studies performed in adult patients with acute ischemic stroke consistently reported that impairment in semantic fluency was related to structural damage to frontal, temporal and parietal cortical regions.^40^ Verbal memory, on the other hand, involves the medial temporal lobe for memory formation and consolidation, and the frontal and parietal cortices for retrieval.^43^ The overlap of neural regions involved in both semantic fluency and verbal memory suggests the existence of a common cortical substrate. In line with this hypothesis, we found that pediatric MS patients belonging to the MVS phenotype showed reduced cortical GM volume compared to HC. In addition, the fMRI analysis revealed decreased RS FC within the frontoparietal network in patients belonging to this cognitive phenotype. This result is consistent with previous studies showing reduced RS FC in the frontoparietal network in cognitively impaired adult^44^ and pediatric^7^ patients with MS. The frontoparietal network is highly integrated with other networks and serves to quickly instantiate new task states through flexible interaction with other processing and control systems.^45^, ^46^ It plays a crucial role in multiple cognitive abilities, including the retrieval of lexical information from long‐term memory.^47^

The third cluster, MI, comprised 18 pediatric MS patients representing 25% of the sample. Compared to patients with PC, patients with MI exhibited lower scores on all cognitive tests except the phonemic fluency test, lower intelligence quotient scores and more severe physical disability. The cognitive and clinical profile of these patients reflects the diffuse brain structural damage observed on MRI, affecting all brain regions examined except the hippocampus. Moreover, patients with MI exhibited a significant deterioration of structural connectivity in four of seven cortical networks (somatomotor network, dorsal attention network, limbic network, and frontoparietal network) compared to patients with PC. Compared to HC, they exhibited reduced RS FC degree within the frontoparietal network. This large‐scale network may be particularly susceptible to the pathophysiological effects of MS^44^ and to the failure of WM maturational changes due to pediatric onset,^48^ potentially affecting the development of cognitive abilities.^3^ Our findings support this notion, as we observed reduced RS FC within the frontoparietal network in both MI and MVS phenotypes, underscoring the key role of frontoparietal network integrity in explaining MS‐related cognitive impairment.^7^, ^35^, ^44^

This study has some limitations. First, the cross‐sectional design precludes examining the stability of cognitive phenotypes across different time points and their evolution over time. Second, although the cohort of patients enrolled in this study is relatively large considering the rarity of pediatric MS and the difficulty to perform MRI studies, the statistical power of subgroup analysis for each cluster may be limited due to the sample size. For the same reasons, an independent validation cohort was not available. Fourth, brain images were acquired using two different scanners. Although the z‐scores of MRI metrics were calculated according to the distributions for each scanner, it is possible that the influence of different imaging parameters was not fully accounted for. To address this issue, we used the same parameters for fMRI sequence. Finally, the current results depend heavily on the statistical approach. Therefore, future studies should assess the reproducibility of these findings from independent datasets.

Our study provides insights into cognitive phenotypes in pediatric MS as well as associated clinical and MRI markers. We observed that two of the three neuropsychological profiles align with the conventional notions of intact cognition and global cognitive impairment. Of note, a third group, characterized by mild deficits in verbal memory and semantic fluency, introduced a novel cognitive phenotype that challenged the dichotomous view of cognitive function in pediatric MS. As the field of MS moves toward establishing more meaningful neuropsychological taxonomies, identifying distinct cognitive phenotypes and understanding their underlying neurobiology could contribute to the development of more tailored diagnostic and therapeutic approaches. By including a comprehensive neuropsychological evaluation that allows for the detection of marked and subtle cognitive deficits, clinicians can refine their assessment and identify who may benefit from targeted interventions. Cognitive training programs that focus on specific cognitive abilities according to the patient's cognitive phenotype may offer a promising way to mitigate future cognitive decline.^49^

## Author Contributions

*Concept and design*: Filippi, Rocca. *Acquisition, analysis, or interpretation of data*: Mistri, Margoni, Pagani, Valsasina, Meani, Moiola, Filippi, Rocca. *Drafting of the manuscript*: Mistri, Margoni, Pagani, Valsasina, Meani, Filippi, Rocca. *Critical revision of the manuscript for important intellectual content*: Mistri, Margoni, Pagani, Valsasina, Meani, Moiola, Filippi, Rocca. *Statistical analysis*: Mistri, Meani. *Supervision*: Filippi, Rocca.

## Conflict of Interest Statement

D. Mistri, E. Pagani, P. Valsasina, and A. Meani have nothing to disclose. M. Margoni reports grants and personal fees from Sanofi Genzyme, Merck Serono, Novartis, and Almiral. L. Moiola received compensation for speaking activities, travel grant, and participation in advisory board from Biogen, Bristol‐Myers Squibb, Novartis, Roche, Sanofi‐Genzyme, Merck‐Serono, Biogen, and Alexion. M. Filippi is Editor‐in‐Chief of the Journal of Neurology, Associate Editor of Human Brain Mapping, Neurological Sciences, and Radiology; received compensation for consulting services from Alexion, Almirall, Biogen, Merck, Novartis, Roche, and Sanofi; speaking activities from Bayer, Biogen, Celgene, Chiesi Italia SpA, Eli Lilly, Genzyme, Janssen, Merck‐Serono, Neopharmed Gentili, Novartis, Novo Nordisk, Roche, Sanofi, Takeda, and TEVA; participation in Advisory Boards for Alexion, Biogen, Bristol‐Myers Squibb, Merck, Novartis, Roche, Sanofi, Sanofi‐Aventis, Sanofi‐Genzyme, Takeda; scientific direction of educational events for Biogen, Merck, Roche, Celgene, Bristol‐Myers Squibb, Lilly, Novartis, and Sanofi‐Genzyme; he receives research support from Biogen Idec, Merck‐Serono, Novartis, Roche, the Italian Ministry of Health, the Italian Ministry of University and Research, and Fondazione Italiana Sclerosi Multipla. M.A. Rocca received consulting fees from Biogen, Bristol Myers Squibb, Eli Lilly, Janssen, Roche; and speaker honoraria from AstraZaneca, Biogen, Bristol Myers Squibb, Bromatech, Celgene, Genzyme, Horizon Therapeutics Italy, Merck Serono SpA, Novartis, Roche, Sanofi, and Teva. She receives research support from the MS Society of Canada, the Italian Ministry of Health, the Italian Ministry of University and Research, and Fondazione Italiana Sclerosi Multipla. She is associate editor for multiple sclerosis and related disorders.

## Funding Information

The authors have not declared a specific grant for this research from any funding agency in the public, commercial, or not‐for‐profit sectors.

## Data Availability

The anonymized dataset used and analyzed during the current study is available from the corresponding author upon reasonable request.
